# Supplementary material for: TGF-β drives the conversion of conventional NK cells into uterine tissue-resident NK cells to support murine pregnancy
Source: eLife. 2026 Jul 8;15:RP109878. doi: 10.7554/eLife.109878 (PMC13345626; doi:10.7554/eLife.109878)
Supplement: Peer review history

# Editor report: eLife Assessment

**DOI:** 10.7554/eLife.109878.3.sa0

**Authors:** Tomohiro Kurosaki

**Affiliations:** The University of Osaka Japan

**Keywords:** Convincing, Important

The importance of uterine natural killer (NK) cells in reproductive success has been demonstrated in mice and humans; however, it is still unclear how uterine NK cells are developed. In this **important** manuscript, the authors provide **convincing** evidence that TGF-b signaling in NK cells supports normal pregnancy in mice by the conversion of conventional NK cells into uterine tissue-resident NK cells. Previous concerns have been addressed in this revised version.

# Referee report: Reviewer #1 (Public review)

**DOI:** 10.7554/eLife.109878.3.sa1

This is an excellent paper from Dr. Yokoyama and colleagues. The experiments are technically demanding, given the very low cell numbers and the challenges of working with implantation sites at gestational days 6.5, 10.5, and 14.5. Overall, the impact of TGF-β receptor II deficiency in the NK lineage on uterine trNK cell numbers and litter size is convincing, and the authors' conclusions are well supported by the data. Less convincing, however, is the claim that the decrease in trNK cells is compensated by an increase in cNK cells; rather, the absence of TGF-β receptor II appears to result in an overall reduction of NK/ILC1 cells.

Comments on revised version:

I thank the authors for addressing all my comments from my initial review.

# Referee report: Reviewer #2 (Public review)

**DOI:** 10.7554/eLife.109878.3.sa2

In their manuscript "TGF-β drives the conversion of conventional NK cells into uterine tissue-resident NK cells to support murine pregnancy", Yokoyama and colleagues investigate the role of Tgfbr2 expression by NK cells in the formation of tissue-resident uterine NK cells and subsequent importance in murine pregnancy. By transferring congenic splenic conventional NK cells into pregnant mice, they show conversion of circulating NK cells into uterine ivCD45 negative tissue-resident NK cells. When interfering with the formation of uterine trNK cells, spiral artery remodelling was impaired, fetal resorption rates were increased, and litter sizes were reduced.

Generally, this is a research topic of high interest, yet the manuscript is lacking detailed mechanistical insights and some questions remain open. At the current state, the data represent an interesting characterisation of the Tgfbr2-fl/fl Ncr1-Cre mice in pregnancy, but considering (1) the recent publication by the group (Ref#17) on the role of Eomes+ cNK cells during pregnancy, (2) the previously described role of Tgfbr2 and autocrine TGFb expression for uterine NK cell differentiation in virgin mice (also cited by the authors), and (3) the well-known relevance of uterine NK cells during pregnancy, additional experiments addressing the specific role of Tgfb during pregnancy would help to improve novelty and significance of the manuscript.

Comments on revised version:

In their revised version of the manuscript and their point-by-point response, the authors have very carefully addressed and discussed all of our concerns and suggestions.

# Author comment: Author response

**DOI:** 10.7554/eLife.109878.3.sa3

**Authors:** Josselyn D Barahona, Liping Yang, D Michael Nelson, Wayne M Yokoyama

**Affiliations:** Washington University in St. Louis St Louis United States; Washington University School of Medicine St Louis United States; Washington University in St. Louis St Louis United States; Washington University in St. Louis St Louis United States

The following is the authors’ response to the original reviews.

**Reviewer #1 (Public review):**

(1) Figure 1A and B: Although a trend is evident, it does not appear that the absolute number of cNK cells at day 14 is significantly changed from day 6.5?

We thank the reviewer for this careful observation. We had not originally performed a statistical comparison between the number of cNK cells present at gds 6.5 and 14.5. We have now conducted the appropriate statistical analysis for this dataset and found that the absolute number of cNK cells at day 14.5 is in fact significantly different from day 6.5 (p = 0.0005; unpaired *t test*, Mann-Whitney correction). The figure and corresponding legend have been updated to reflect this analysis. Please see Figure 1B:

“Statistics were calculated using unpaired *t tests* with the Mann-Whitney correction. Error bars indicate SEM; *** *p* < 0.001.”

(2) Figure 2E: The authors state, "This reduction of uterine trNK cells was accompanied by a concomitant increase in the absolute number and frequency of CD49b+Eomes+ cNK cells within the pregnant uterus of TGF-βRIINcr1Δ dams (Figure 2 D, E). The number of cNK cells appears relatively low (visually ~1,000-1,300), and although the difference is statistically significant, its physiological relevance is unclear. More importantly, this modest increase does not correlate with the marked decrease in trNK and ILC1 populations, as cNK cells do not appear to accumulate. In my opinion, the conclusion "Collectively, these findings indicate that a TGF-β-driven differentiation pathway directs the conversion of peripheral cNK cells into uterine trNK cells during murine pregnancy" should be slightly toned down.

We thank both reviewers for this suggestion. Regarding the absence of cNK cell accumulation in the absence of TGF-β signaling, we suggest that this may be related to the normal passage of cNK cells circulating in the placenta, i.e., these cells may not have acquired signals to remain in the uterus and are simply continuing to pass through and not accumulating. Nonetheless, we have rephrased our wording in to address this concern as follows:

“This reduction of uterine trNK cells was accompanied by a small increase in the absolute number and frequency of CD49b^+^ Eomes^+^ cNK cells within the pregnant uterus of TGF-βRII^Ncr1∆^ dams (Figure 2 D, E). Collectively, these findings suggest that a TGF-β–driven differentiation pathway directs the conversion of peripheral cNK cells into uterine trNK cells during murine pregnancy.”

“The absence of cNK cell accumulation in the gravid uterus in the setting of impaired TGF-β signaling suggests a defect in tissue retention rather than recruitment. In the absence of TGF-β–mediated cues, circulating cNK cells that enter the uterine vasculature may fail to acquire the molecular programs required for residency and instead continue to transit through the tissue. This is consistent with a model in which TGF-β signaling promotes not only phenotypic conversion but also the acquisition of retention signals necessary for persistence within the uterine microenvironment, reinforcing that acquisition of tissue-residency in the gravid uterus is an actively instructed process [29,32].”

(3) Figures 2-4: It is unclear whether the littermate controls are floxed mice or floxhet-Ncr1iCre mice? This distinction is important, as Ncr1iCre expression itself could potentially lead to a phenotype.

To address these concerns, we characterized the uterine innate lymphoid cell compartment in the pregnant uterus of Ncr1^icre^ dams at gestational day 6.5. We did not observe a difference in the absolute number and frequency of trNK cells, cNK cells, and ILC1s in the gravid uterus of Ncr1^icre^ dams compared to wildtype CD45.1 C57BL/6 mice. Additionally, the number of implantation sites and resorption rates in Ncr1^icre^ dams was comparable to wildtype CD45.1 C57BL/6 mice. Together these data indicate that Ncr1^icre^ expression itself does not influence the phenotype we report in TGF-βRII^Ncr1∆^ dams. These additional findings have been included in Supplementary Figure 1 and in the text as follows:

“To ensure we exclude a confounding effect of Ncr1^iCre^ expression, we profiled the uterine innate lymphoid compartment in pregnant Ncr1^iCre^ dams at gestational day 6.5. No differences were observed in the absolute number of trNK cells, cNK cells, or ILC1s relative to wildtype controls (Figure S1 A-D), and implantation site number and resorption rates were likewise unchanged (Figure S1 E-F). These data indicate that Ncr1^iCre^ expression alone does not perturb uterine ILC composition or early pregnancy outcomes.”

**Reviewer #1 (Recommendations for the authors):**

(1) Figure 1C &D: The adoptive transfer experiment is convincing. As a minor point, why is the gate setting for Eomes different between panels 1C and 1D?

To clarify the phenotype of the adoptively transferred cNK cells, we included two additional gates depicting the expression of CD49a and CD49b in unlabeled (non-vascular) trNK cells and cNK cells in the pregnant uterus Please see the revised Figure 1C and revised figure legend:

“(C) Concatenated flow plots of implantation sites showing that adoptively transferred cNK cells in pregnant uterus of wildtype dams upregulate CD49a and down regulate CD49b by gd 10.5, acquiring a CD49a^+^ CD49b^-^ Eomes^+^ phenotype characteristic of uterine trNK cells (C57BL/6 dams *n*=4). Here, 2.5x10^6^ CD45.2^+^ CD3^-^ CD19^-^ NK1.1^+^ NKp46^+^ CD49b^+^ splenic cNK cells were adoptively transferred into pregnant C57BL/6-CD45.1 dams at gd 0.5, and the receptor profile of these cells was subsequently assessed at gd 10.5. Gating strategy: Live, Single Cells; CD3^-^ CD19^-^ CD45.1^-^ CD45.2–PE-Cy7^-^ CD45.2–PE^+^ NK1.1^+^ NKp46^+^ cells.”

(2) Figure 3: Has the pup ratio male/female changed?

We did not observe a statistically significant difference in the female-to-male pup ratio between groups.

**Reviewer #2 (Public review):**

(1) The authors suggest cNK extravasation and local differentiation into iv- trNK. Can it be estimated how much this process contributes to the trNK pool vs. a potential local proliferation of already existing trNK? How do absolute numbers of CD49a+ Eomes+ trNK change during pregnancies? (In Figure 1A, the cell numbers of CD49a+ Eomes+ trNK seem to go down dramatically between gd 6.5 and 14.5). The plot in 1B could also include absolute numbers of ILC1s and trNKs. Would recruited cNK cells compensate for a potential loss of CD49a+ Eomes+ trNK?

Our prior work as well as others have tracked the changes in uterine trNK cells, cNK cells, and ILC1s over the course of murine pregnancy. Consistent with these studies, the absolute number of uterine CD49a^+^ Eomes^+^ trNK cells peaks during early pregnancy (roughly between gds 5.5 7.5) and subsequently declines until term. The decrease in uterine trNK cells between gd 6.5 and gd 14.5 observed in Figure 1A is therefore consistent with the known physiological contraction of the decidual NK compartment as pregnancy progresses. Thus, it is unlikely that cNK cells recruited within the uterine tissue compensate for the loss of CD49a^+^ Eomes^+^ trNK cells observed. To address the reviewer’s request, we have now included the absolute number of uterine trNK cells and ILC1s in Figure 1–please see updated Figure 1C and D and corresponding figure legend (provided below). With respect to the relative contribution of cNK cells extravasation vs local proliferation of trNK cells, our data do not allow us to quantitatively distinguish between these mechanisms. Moreover, previous studies have demonstrated that uterine trNK cells express Ki67, suggesting that they exhibit proliferative activity during this period. Thus, we hypothesize that both local proliferation of existing trNK cells and recruitment of circulating cNK cells contribute to the population of uterine trNK cells during early pregnancy.

“(C) Concatenated flow plots of implantation sites showing that adoptively transferred cNK cells in pregnant uterus of wildtype dams upregulate CD49a and down regulate CD49b by gd 10.5, acquiring a CD49a^+^ CD49b^-^ Eomes^+^ phenotype characteristic of uterine trNK cells (C57BL/6 dams *n*=4). Here, 2.5x10^6^ CD45.2^+^ CD3^-^ CD19^-^ NK1.1^+^ NKp46^+^ CD49b^+^ splenic cNK cells were adoptively transferred into pregnant C57BL/6-CD45.1 dams at gd 0.5, and the receptor profile of these cells was subsequently assessed at gd 10.5. Gating strategy: Live, Single Cells; CD3^-^ CD19^-^ CD45.1^-^ CD45.2–PE-Cy7^-^ CD45.2–PE^+^ NK1.1^+^ NKp46^+^ cells. (D) Proportion of uterine ILC subsets derived from adoptively transferred splenic cNK cells in the pregnant uterus of wildtype dams. Statistics were calculated using unpaired *t tests* with the Mann-Whitney correction. Error bars indicate SEM; ***p < 0.001.”

Barahona, J.D., Yang, L. and Yokoyama, W.M., 2025. Eomesodermin defines uterine NK cells crucial for pregnancy success in mice. The Journal of Immunology, 214(10), pp.2549-2556.

Filipovic, I., Chiossone, L., Vacca, P., Hamilton, R.S., Ingegnere, T., Doisne, J.M., Hawkes, D.A., Mingari, M.C., Sharkey, A.M., Moretta, L. and Colucci, F., 2018. Molecular definition of group 1 innate lymphoid cells in the mouse uterus. Nature Communications, 9(1), p.4492.

(2) Figure 1C: 2.5 Mio cNK cells have been transferred, but only very few cells can be detected within the uterus (concatenated FACS plot shown). What may represent the limit to generate uterine trNK out of cNK? Is the niche supporting cNK-trNK differentiation limited? Is it only a specific subset of (splenic) cNK capable of differentiating into trNK? Is gd 0.5 the optimal timepoint for the transfer? Is there continuous recruitment of cNK into the uterus and differentiation into trNK, or is it enhanced at specific timepoints of pregnancy? Could there be local proliferation of cNK-derived trNK? This could be studied by proliferation dye dilution of WT cNK cells in this transfer-setup.

We recognize that transferring cNK cells at gestational day 0.5–prior to placental formation–may partially account for the low uterine reconstitution observed. At this time point, the local signals necessary for efficient recruitment and retention of cNK cells in the uterus may not yet be fully established, potentially resulting in preferential homing to peripheral tissues such as the spleen and liver. Consistent with this possibility, we do observe a robust population of adoptively transferred cNK cells in the spleen and liver of our pregnant dams. We decided to transfer cNK cells at gestational day 0.5 to ensure that the cells were present at throughout most of early pregnancy, particularly during implantation and the initial stages of decidualization. We also did not transfer cells before mating to minimize the number of mice that did not get pregnant. Additionally, performing the transfer at this early time point minimized repeated manipulation of pregnant dams, as procedural stress itself has been shown to affect physiological processes of gestation and could thereby confound the pregnancy outcomes we were assessing. Furthermore, Filipovic et al. 2018 previously showed that both trNK cells and cNK cells in the pregnant uterus expressed Ki67 at gestational 9.5, suggesting that there could be local proliferation of cNK-derived trNK cells in the gravid uterus that could limit the migration of circulating cNK cells into this microenvironment. We have discussed in more depth in our discussion section as follows:

“Interestingly, the inability to fully reconstitute the uterine trNK cell compartment following adoptive transfer suggests that only a subset of circulating cNK cells may be capable of differentiating into trNK cells during pregnancy, or alternatively that trNK cells already present in the virgin uterus may undergo in situ proliferation in the gravid uterus. Previous studies from our lab as well as others show that trNK cells within the pregnant murine uterus express marked levels of Ki67, supporting a model in which local proliferation of uterine trNK cells is a major contributor to the uterine trNK cell pool during pregnancy [7,32]. Prior studies have also described hematopoietic precursors within endometrial and decidual tissues that generate uterine trNK cells, suggesting that the compartment may be also sustained by local precursor differentiation [33-35]. Together, these findings suggest that uterine trNK cell ontogeny may be more complex than a single-source model and raise the possibility that distinct developmental pathways may operate at different stages of reproductive life. Therefore, defining the relative contribution and developmental timing of hematogenous versus locally maintained sources in vivo could provide relevant insights into the developmental trajectories and transcriptional programs that underlie decidual NK cell heterogeneity.”

Zhai, Q.Y., Wang, J.J., Tian, Y., Liu, X. and Song, Z., 2020. Review of psychological stress on oocyte and early embryonic development in female mice. Reproductive Biology and Endocrinology, 18(1), p.101.

Wiebold, J.L., Stanfield, P.H., Becker, W.C. and Hillers, J.K., 1986. The effect of restraint stress in early pregnancy in mice. Reproduction, 78(1), pp.185-192.

Sánchez-Rubio, M., Abarzúa-Catalán, L., Del Valle, A., Méndez-Ruette, M., Salazar, N., Sigala, J., Sandoval, S., Godoy, M.I., Luarte, A., Monteiro, L.J. and Romero, R., 2024. Maternal stress during pregnancy alters circulating small extracellular vesicles and enhances their targeting to the placenta and fetus. Biological Research, 57(1), p.70.

Filipovic, I., Chiossone, L., Vacca, P., Hamilton, R.S., Ingegnere, T., Doisne, J.M., Hawkes, D.A., Mingari, M.C., Sharkey, A.M., Moretta, L. and Colucci, F., 2018. Molecular definition of group 1 innate lymphoid cells in the mouse uterus. Nature Communications, 9(1), p.4492.

(3) The authors should consider inducible Tgfbr2 deletion (e.g. with Tamoxifen-inducible Cre) to enable development of the uterine NK compartment in virgin mice and only ablate trNK differentiation during pregnancy. This could help to estimate the turnover of cNK into trNK, or to understand if constant cNK recruitment is required to form the uterine trNK compartment during pregnancy.

Thank you for this suggestion. We did initially consider incorporating a mouse model with a tamoxifen-inducible deletion of the TGF-βRII to examine the differentiation of peripheral cNK cells into uterine trNK cells more precisely. However, the administration of tamoxifen during murine pregnancy has well-established deleterious effects on implantation, fetal viability, and placentation, which would confound our interpretations of any adverse pregnancy outcome observed in our studies. Because our goal was to assess NK cell-specific contributions to murine gestation without introducing additional pregnancy-related perturbations, we elected to use an Ncr1^iCre^ – based mouse model in our studies.

Ved, N., Curran, A., Ashcroft, F.M. and Sparrow, D.B., 2019. Tamoxifen administration in pregnant mice can be deleterious to both mother and embryo. Laboratory animals, 53(6), pp.630-633.

Sun, M.R., Steward, A.C., Sweet, E.A., Martin, A.A. and Lipinski, R.J., 2021. Developmental malformations resulting from high-dose maternal tamoxifen exposure in the mouse. PLoS One, 16(8), p.e0256299.

Ilchuk, L.A., Stavskaya, N.I., Varlamova, E.A., Khamidullina, A.I., Tatarskiy, V.V., Mogila, V.A., Kolbutova, K.B., Bogdan, S.A., Sheremetov, A.M., Baulin, A.N. and Filatova, I.A., 2022. Limitations of tamoxifen application for in vivo genome editing using Cre/ERT2 system. International Journal of Molecular Sciences, 23(22), p.14077.

(4) Did the authors consider transfer of Tgfbr2-floxed Ncr1-Cre cNK in the same setup as in Fig. 1C? This experiment could confirm the requirement of Tgfbr-dependent signaling for cNK to trNK conversion during pregnancy versus effects of Tgfb signals on trNK numbers in the uterus at steady state (before pregnancy).

We thank the reviewer for this mechanistically insightful suggestion. We did consider performing reciprocal transfer experiments using TGF-βRII^fl/fl^ Ncr1^icre^ cNK cells in the same adoptive transfer system as in Figure 1C. Our current adoptive transfer experiments already directly address this question. Transfer of congenically labeled wild-type splenic cNK cells into TGF-βRII^Ncr1Δ^ dams at gestational day 0.5 resulted in partial reconstitution of the uterine trNK compartment and, importantly, this was sufficient to rescue the adverse pregnancy outcomes observed at midgestation. These findings indicate that TGF-β–competent cNK cells can differentiate and function appropriately within the pregnant uterine environment, supporting a requirement for TGF-β–dependent signaling in cNK-to-trNK conversion during pregnancy. Because restoration of TGF-β–sufficient cNK cells rescues these pregnancy outcomes, we believe this experiment functionally demonstrates the importance of TGF-β signaling in this process and therefore did not pursue reciprocal transfer of TGF-βRII–deficient cNK cells.

“Partial reconstitution of uterine trNK cells restores midgestational pregnancy outcomes in TGF-βRII^Ncr1∆^ dams

To determine whether restoring uterine trNK cells could rescue the midgestational pregnancy defects observed in TGF-βRII^Ncr1∆^ dams, we adoptively transferred wildtype, congenically labeled splenic cNK cells into pregnant TGF-βRII^Ncr1∆^ dams at gd 0.5. By gd 10.5, donor cNK cells were detected in the pregnant uterus, where a subset upregulated CD49a and downregulated CD49b, consistent with acquisition of a uterine trNK cell phenotype (Figure 5 A). However, adoptively transferred splenic cNK cells only partially reconstituted the uterine trNK cell population in the gravid uterus of TGF-βRII^Ncr1∆^ dams, as evidenced by reduced absolute number and frequency of donor-derived trNK cells in reconstituted TGF-βRII^Ncr1∆^ dams (Figure 5 A-C). Notably, this partial reconstitution was sufficient to rescue the gestational defects caused by impaired TGF-β–mediated uterine trNK cell differentiation. Reconstituted TGF- βRII^Ncr1∆^ dams exhibited implantation site numbers and fetal resorption rates at gd 10.5 comparable to those observed in littermate controls (Figure 5 D, E). Together, these findings suggest that even partial restoration of the uterine trNK cell in pregnant TGF-βRII^Ncr1∆^ dams is sufficient to restore pregnancy outcomes at midgestation, supporting a central role for uterine trNK cells as the principal NK cell subset required for successful murine pregnancy.”

(5) Figures 2D/E: The authors should state that ILC1s are reduced in the virgin uterus of female Tgfbr2-floxed or Tgfb1-floxed Ncr1-Cre mice and cite the relevant work (the Ref #29 discussed in this context did not show that?). It would be helpful to include an analysis of all three uterine ILC subsets in steady state. This could help to answer the question if the cNK cell changes are pregnancy-specific or a general phenomenon in Tgfbr2-floxed Ncr1-Cre mice.

We thank the reviewer for this important comment and for noting the miscitation. We regret the error and have corrected the reference in the revised manuscript to cite the appropriate study demonstrating reduced ILC1s in the virgin uterus of Tgfb1^fl/fl^ Ncr1^iCre^ mice {Sparano, C. et al. 2024. Autocrine TGF-β1 drives tissue-specific differentiation and function of resident NK cells. Journal of Experimental Medicine, 222(3), p.e20240930}. Please see Line 148. Importantly, the steady-state ILC compartment in virgin Tgfb1^fl/fl^ Ncr1^iCre^ mice has already been carefully characterized in the previously published work, including analysis of all three uterine ILC subsets. Because the steady-state uterine ILC landscape in this mouse model has already been established by Sparano, C. et al. 2024, our study focuses specifically on the pregnancy-associated changes in the uterine ILC landscape occurring in the absence of TGF-β signaling in *Ncr1*-expressing cells and their subsequent effects on gestational outcomes. In the absence of TGF-β signaling there appears to be a higher frequency of cNK cells in both the virgin uterus and pregnant uterus, suggesting that this is more of a general phenomenon.

“However, in the pregnant uterus, CD49a^+^ Eomes^-^ ILC1s were markedly reduced in implantation sites of TGF-βRII^Ncr1∆^ dams, paralleling the reduction of ILC1s previously reported in the virgin uterus of TGF-βRII^Ncr1∆^ female mice [26].”

(6) Figure 2E: Please phrase more carefully about the "concomitant increase" of cNKs, since this increase is much less pronounced compared to the very strong reduction (absence) of trNKs in Tgfbr2-floxed Ncr1-Cre mice. Do the authors suggest that cNKs are halted at this stage and cannot differentiate into trNK, based on these data?

We thank both reviewers for this suggestion, and we have rephrased our wording to address this concern as follows:

“This reduction of uterine trNK cells was accompanied by a small increase in the absolute number and frequency of CD49b^+^ Eomes^+^ cNK cells within the pregnant uterus of TGF-βRII^Ncr1∆^ dams (Figure 2 D, E). Collectively, these findings suggest that a TGF-β–driven differentiation pathway directs the conversion of peripheral cNK cells into uterine trNK cells during murine pregnancy.”

Please also see our response to Reviewer #1, Comment #2.

(7) Can the reduced litter size and the abnormal spiral artery formation be rescued by transfer of WT cNK into Tgfbr2-floxed Ncr1-Cre mice?

We thank the reviewers for this interesting question. In subsequent experiments, we transferred congenically labeled, splenic cNK cells from wildtype female mice into TGF-βRII^Ncr1∆^ dams at gestational day 0.5. We only observed partial reconstitution of uterine trNK cell population; however, the number of viable implantation sites and resorption rates in reconstituted TGF-βRII^Ncr1∆^ dams were comparable to the number of viable implantation sites and resorption rates in HBSS-treated littermate controls at gestational day 10.5. Given that partial reconstitution of the uterine trNK cell compartment in reconstituted TGF-βRII^Ncr1∆^ dams was sufficient to rescue the defects in implantation site number and fetal resorption rates observed at midgestation, we hypothesize that this level of restoration may permit patrial but functionally sufficient spiral artery remodeling to reestablish maternal-fetal blood flow adequate to support fetal viability, although spiral artery remodeling was not directly assessed in this transfer study.

“Partial reconstitution of uterine trNK cells restores midgestational pregnancy outcomes in TGF-βRII^Ncr1∆^ dams

To determine whether restoring uterine trNK cells could rescue the midgestational pregnancy defects observed in TGF-βRII^cr1∆^ dams, we adoptively transferred wildtype, congenically labeled splenic cNK cells into pregnant TGF-βRII^Ncr1∆^ dams at gd 0.5. By gd 10.5, donor cNK cells were detected in the pregnant uterus, where a subset upregulated CD49a and downregulated CD49b, consistent with acquisition of a uterine trNK cell phenotype (Figure 5 A). However, adoptively transferred splenic cNK cells only partially reconstituted the uterine trNK cell population in the gravid uterus of TGF-βRII^Ncr1∆^ dams, as evidenced by reduced absolute number and frequency of donor-derived trNK cells in reconstituted TGF-βRII^Ncr1∆^ dams (Figure 5 A-C). Notably, this partial reconstitution was sufficient to rescue the gestational defects caused by impaired TGF-β–mediated uterine trNK cell differentiation. Reconstituted TGF-βRII^Ncr1∆^ dams exhibited implantation site numbers and fetal resorption rates at gd 10.5 comparable to those observed in littermate controls (Figure 5 D, E). Together, these findings suggest that even partial restoration of the uterine trNK cell in pregnant TGF-βRII^Ncr1∆^ dams is sufficient to restore pregnancy outcomes at midgestation, supporting a central role for uterine trNK cells as the principal NK cell subset required for successful murine pregnancy.”

**Reviewer #2 (Recommendations for the authors):**

(1) Figure 1C: The shown gate seems to "cut" into the CD49b staining; staining for all transferred cells should be shown; have cNK cells been stained in parallel with the same panel to provide a positive and compensation control?

To clarify the phenotype of the adoptively transferred cNK cells, we included two additional gates depicting the expression of CD49a and CD49b in unlabeled (non-vascular) trNK cells and cNK cells in the pregnant uterus Please see the revised Figure 1C.

“(C) Concatenated flow plots of implantation sites showing that adoptively transferred cNK cells in pregnant uterus of wildtype dams upregulate CD49a and down regulate CD49b by gd 10.5, acquiring a CD49a^+^ CD49b^-^ Eomes^+^ phenotype characteristic of uterine trNK cells (C57BL/6 dams *n*=4). Here, 2.5x10^6^ CD45.2^+^ CD3^-^ CD19^-^ NK1.1^+^ NKp46^+^ CD49b^+^ splenic cNK cells were adoptively transferred into pregnant C57BL/6-CD45.1 dams at gd 0.5, and the receptor profile of these cells was subsequently assessed at gd 10.5. Gating strategy: Live, Single Cells; CD3^-^ CD19^-^ CD45.1^-^ CD45.2–PE-Cy7^-^ CD45.2–PE^+^ NK1.1^+^ NKp46^+^ cells.”

(2) Figure 2A: The authors could include an isotype control or a staining in a genetic knockout as a control staining.

Thank you for this suggestion. As suggested, we included staining in a genetic TGF-βRII^Ncr1∆^ knockout as additional control staining. Please see the revised Figure 2A.

“Representative histograms depicting TGF-β Receptor II expression on splenic NK cells from virgin TGF-βRII^Ncr1∆^ and wildtype mice as well as splenic and uterine NK cell subsets from pregnant wildtype mice at gd 10.5 (virgin TGF-βRII^Ncr1∆^ mice, *n*=2; virgin mice: C57BL/6, *n*=5; gd 10.5: C57BL/6 dams, *n*=8, implantation sites *n*=8). MFI, median fluorescent intensity. Gating strategy: Live, Single Cells; CD3^-^ CD19^-^ CD45.1^-^ CD45.2^+^ NK1.1^+^ NKp46^+^ cells.”
